# Proposing the ValvUS approach: integrating bedside tests and ultrasonography for severe valvular heart disease diagnosis

**DOI:** 10.31744/einstein_journal/2026RW1487

**Published:** 2025-12-01

**Authors:** Tarso Augusto Duenhas Accorsi, Henrique Barbosa Ribeiro, Ranna Santos Pessoa, Renato Paladino Nemoto, Wilson Mathias Junior, Philippe Pibarot, Flavio Tarasoutchi

**Affiliations:** 1 Hospital das Clínicas Faculdade de Medicina Universidade de São Paulo São Paulo SP Brazil Instituto do Coração (InCor), Hospital das Clínicas, Faculdade de Medicina, Universidade de São Paulo, São Paulo, SP, Brazil.; 2 Department of Medicine Québec Heart & Lung Institute Laval University Québec City Québec Canada Department of Medicine, Québec Heart & Lung Institute, Laval University, Québec City, Québec, Canada.

**Keywords:** Heart valve diseases, Aortic valve disease, Mitral valve, Physical examination, Ultrasonography, Point-of-care testing, Point-of-care systems

## Abstract

Valvular heart disease is increasingly prevalent, and bedside confirmation or exclusion of severe disease is needed to enable a rapid and cost-effective diagnostic workup. The physical examination skills of clinicians are insufficient for accurate diagnosis, making complementary tests generally necessary. Despite being commonly requested, electrocardiography and chest radiography present low positive and negative likelihood ratios. Incipient studies involving artificial intelligence have shown promising opportunities to support the diagnosis. In addition, solid current evidence demonstrates that point-of-care ultrasound enhances bedside diagnosis of several cardiovascular conditions. Echocardiographic skills can be acquired after only a few hours of training, which encourages routine bedside use with handling equipment. Despite the routine use of sonography in emergencies, large-scale simplified screening protocols for valvular disease remain lacking. Therefore, improving the accuracy of valvular heart disease diagnosis by integrating all bedside modalities needs to be better understood. We propose a simple, reproducible five-step point-of-care ultrasound protocol for diagnosing valvular heart disease (the ValvUS approach), applicable to all patients. The proposed visual assessment involves evaluating valvular movement, thickness, regurgitant flow, aliasing, and chamber dimensions. This evaluation should be interpreted in the context of traditional clinical probability to ensure the most accurate bedside diagnosis. Typical findings of severe valvular disease on electrocardiography and chest radiography, and particularly on point-of-care ultrasound, may improve the accuracy of bedside diagnosis after clinical assessment in the near future.

## INTRODUCTION

Valvular heart disease (VHD) is increasingly prevalent among outpatients, particularly in the older population, and the final diagnosis is typically established by comprehensive echocardiography following a clinical suspicion.^( 1 )^ Most patients present with non-severe VHD, which has no hemodynamic consequences and no specific treatment recommendations.^( 2 )^ However, identifying severe VHD, which represents the advanced phase of the natural history of the disease, is crucial for recognizing at-risk patients and is a cornerstone of management.^( 3 )^ Bedside confirmation or exclusion of severe VHD is presumably associated with time and cost savings, improved clinical reasoning, more appropriate treatment, and greater physician-patient confidence.^( 4 )^

Although the best cardiovascular diagnostic reasoning starts with generating hypotheses through clinical assessment and subsequent testing, current medical practices are firmly based on requesting and reviewing imaging tests.^( 5 , 6 )^ However, this modern approach has not been associated with better outcomes and probably decreases practitioners’ appreciation of the diagnostic value of history-taking and physical examination.^( 7 )^ Regarding bedside VHD diagnosis, previous evidence has demonstrated low to moderate positive and negative likelihood ratios (LR) related to murmur detection.^( 8 )^ Likewise, the physical examination skills of clinicians are generally limited for diagnosing VHD, and great heterogeneity exists in the experience of professionals, including clinicians and teachers.^( 9 )^

Solid current evidence suggests that electrocardiography, chest radiography, and point-of-care ultrasound (POCUS) enhance the bedside diagnosis of several cardiovascular conditions, thereby complementing the physical examination.^( 10 )^ Electrocardiography and chest radiography may suggest chamber overload related to severe VHD; however, their diagnostic accuracy is generally limited, with low LR.^( 11 , 12 )^ Echocardiographic skills can be acquired after only a few hours of training, which encourages routine bedside use with handling equipment.^( 13 )^ Large-scale, simplified screening protocols for valvular disease are lacking.^( 14 )^ More recently, artificial intelligence programs have been developed to assist physicians by analyzing complementary tests and highlighting red-flag situations, including VHD.^( 15 )^

However, despite this evidence, the overall diagnostic accuracy of VHD assessment integrating all bedside modalities remains poorly evaluated. This analytical review synthesizes previous and modern diagnostic strategies to propose a practical and effective bedside diagnostic approach for severe VHD, primarily based on POCUS.

## DIAGNOSTIC FLOW

The primary objective of clinical evaluation is to generate diagnostic hypotheses. Cardiology diagnoses are usually probabilistic, and these probabilities are generally conditional.^( 16 )^ Following the estimation of pre-test probabilities, physicians may refine them using specific complementary tests according to each hypothesis. The likelihood ratios represent the capacity of the test to positively or negatively impact the initial hypothesis. Physicians should avoid the “shotgun approach” (requesting multiple tests without appropriate reasoning) and should be familiar with test-specific LR. A positive LR greater than 10 and a negative LR less than 0.1 are more likely to modify the diagnostic hypothesis.^( 17 )^ The initial post-test probability may be sufficient to establish the diagnosis; if not, the diagnostic flow resumes from this point using an additional test.

**Figure 1:**
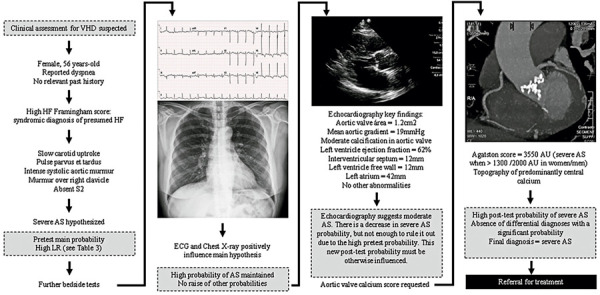
Diagnostic reasoning flow in a patient with suspected aortic stenosis

Regarding severe VHD, the pre-test probability is determined primarily through cardiac auscultation.^( 18 )^ As patient complexity increases, cardiac comorbidities become more common. Paradoxically, simply reviewing an echocardiogram may be insufficient for establishing a valvular diagnosis, underscoring the importance of a structured diagnostic flow. An illustration of the importance of clinical pre-test probability is presented in figure 1 .

## PERIPHERAL PHYSICAL EXAMINATION

The major arterial pulses must be examined to determine their patency, rhythm, waveform, and characteristics. Because arterial pulse waves progressively decrease toward the periphery, the carotid pulse – a central pulse – provides more reliable information regarding left ventricular ejection and aortic valve function.^( 19 )^ A hypokinetic (or hypodynamic) arterial pulse is small and weak and is typically observed in stenosis of the aortic, mitral, or pulmonic valves. Pulsus parvus et tardus (or an anacrotic pulse) refers to a small, delayed systolic peak, usually observed in severe aortic stenosis (AS). A hyperkinetic (or hyperdynamic) arterial pulse is large and strong and is commonly observed in regurgitant valvular disease, which reflects an increased blood volume ejected from the left ventricle. This finding is more pronounced in aortic regurgitation (AR), also known as water hammer pulse *,* than in mitral regurgitation (MR), where it may present only as brisk.^( 20 )^ Other cardiac conditions may also produce this type of pulse; however, interobserver variability has not been well studied, and the LR remains poorly defined.

## AUSCULTATION FLOW

**Figure 2:**
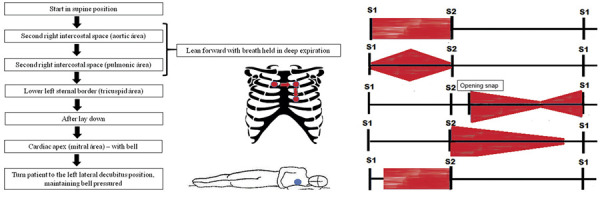
Auscultation flow and murmur configurations

Chest palpation may help detect a valvular thrill, which is usually associated with severe VHD, and can also provide information about ventricular remodeling. Chest percussion has limited clinical value and is rarely emphasized in contemporary practice. A systematic approach to cardiac auscultation is illustrated in figure 2 .

The first split (S1) occurs just before the upstroke of the carotid pulse and is best heard at the apex and along the lower left sternal border, where it is louder than the second split (S2). Because S1 is a high-frequency sound, it is best heard with the diaphragm of the stethoscope.^( 21 - 23 )^ S2 occurs after the peak of the carotid pulse and coincides with the downslope of the pulse. S2 is louder than S1 and is best heard at the left second intercostal space near the sternal border. S2 is best heard in the semi-recumbent position (approximately 30° upright) during quiet inspiration using the stethoscope.^( 21 , 22 , 24 )^ Several abnormalities may affect S1 and S2. The causes of VHD are summarized in table 1 .

**Table 1:** Causes of Abnormalities in S1 and S2

S1 increased intensity	S2 decreased intensity
Mitral stenosis	Aortic stenosis
Tricuspid stenosis	Pulmonary stenosis
S1 decreased intensity	Fixed splitting of S2
Mitral stenosis	Interatrial communication
Splitting of S1	Paradoxical splitting of S2
Tricuspid stenosis	Aortic stenosis

S1: first split; S2: second split.

When a murmur is heard, it must be classified according to the timing within the cardiac cycle, its configuration ( Figure 2 ), and its intensity, using the Levine scale: grade I, very faint and audible only to an expert in a specific position; grade II, soft but audible in all places; grade III, moderately loud without a thrill; grade IV, loud with a thrill; grade V, very loud with a thrill and audible with the stethoscope partially off the chest; and grade VI, audible with the stethoscope completely off the chest.^( 25 )^ To assess the radiation of the murmur, auscultation is performed over the suprasternal notch, axilla, and epigastrium.

Murmurs may also occur in conditions other than VHD. Several bedside maneuvers have been described to aid in identifying the underlying cause of a murmur. Although these maneuvers do not determine disease severity, they may improve diagnostic accuracy when combined with appropriate investigations.^( 26 )^ These maneuvers are summarized in table 2 .^( 22 , 27 )^

**Table 2:** Dynamic auscultation findings

.	
Inspiration	Increased intensity of the murmur in tricuspid regurgitation ( *Rivero Carvallo* sign) Earlier occurrence of the click and murmur in mitral valve prolapse
**Squatting**	Increased intensity of the murmur in mitral and aortic regurgitation Delayed occurrence of the click and murmur in mitral valve prolapse
**Valsalva maneuver**	Decreased intensity of the murmur in aortic and pulmonary stenosis Increased intensity of the murmur in hypertrophic cardiomyopathy
**Standing**	Decreased intensity of the murmur in aortic stenosis, mitral regurgitation, and tricuspid regurgitation **I** ncreased intensity of the murmur in hypertrophic cardiomyopathy
**Handgrip**	Decreased intensity of the murmur in aortic stenosis Increased intensity of the murmur in mitral regurgitation

Suspicion of VHD arises primarily from physical examination, particularly through the detection of the peripheral manifestations of low cardiac output or high regurgitant volume, but most importantly from the presence of a heart murmur.^( 28 )^ An evidence-based summary of physical examination signs, along with their positive and negative LR (LR+ and LR-) for the diagnosis of severe chronic VHD, is shown in table 3 .^( 29 - 33 )^

**Table 3:** Clinical examination findings for the diagnosis of chronic valvular heart disease

Overall examination for detecting valvular heart disease		
	LR+ (95%CI)	LR- (95%CI)
Emergency physicians	14 (10–19)	0.21 (0.14–0.34)
Cardiologists	38 (9.5–154)	0.31 (0.18–0.52)
**Ability to differentiate primary *versus* functional systolic murmur**		
	**LR for Primary (Significant) Systolic Murmur**	
	**LR+ (95%CI)**	**LR- (95%CI)**
Holosystolic murmur	8.7 (2.3–33)	0.19 (0.08–0.43)
Loud murmur	6.5 (2.3–19)	0.08 (0.02–0.31)
Plateau-shaped murmur	4.1 (1.4–12)	0.48 (0.30–0.77)
Loudest at the apex	2.5 (0.58–11)	0.84 (0.65–1.1)
**Physical examination for detecting aortic stenosis**		
	**LR+ (95%CI)**	**LR- (95%CI)**
Slow carotid uptroke	9.2 (3.4–24)	0.56 (0.32–0.8)
Murmur radiating to the right carotid	8.1 (4–16)	0.29 (0.12–0.57)
Reduced or absent S2	7.5 (3.2–17)	0.50 (0.27–0.76)
Murmur over the right clavicle	3.0 (2–4.1)	0.10 (0.02–0.44)
Any systolic murmur	2.6 (1.9–3.5)	0 (0–0.45)
Reduced carotid volume	2.0 (1–3.2)	0.64 (0.34–0.99)
**Combination of findings for aortic stenosis**		
	**LR+ (95%CI) for moderate to severe stenosis**	
Systolic murmur over the right clavicle + 3–4 associated findings	40 (6.6–239)	
Systolic murmur over the right clavicle + 0–2 associated findings	1.8 (0.93–2.9)	
	**LR- (95%CI) for moderate to severe stenosis**	
No systolic murmur over the right clavicle	0.1 (0.02–0.44)	
**Multivariable score for aortic stenosis**		
**Point Score**	**LR+ (95%CI) for severe stenosis**	
14	⇎ (0.6-⇎)	
10–13	8.0 (1.6–46)	
7–9	2.7 (1.0–8.0)	
2–6	0.27 (0.15–0.49)	
**Point Score**	**LR- (95%CI) for severe stenosis**	
0	0.10 (0.01–0.58)	
**Variable**	**Point Score**	
Reduced carotid volume	2	
Slow rate of increase of the carotid pulse	3	
Murmur loudest at the second right intercostal space	2	
Decreased or absent S2	3	
Valve calcification on chest radiography	4	
**Physical examination for detecting mitral regurgitation**		
	**LR+ (95%CI)**	**LR- (95%CI)**
Murmur in the mitral area	3.9 (3.0–5.1)	0.34 (0.23–0.47)
Late or holosystolic murmur	1.8 (1.2–2.5)	0 (0–0.8)
Any murmur during an acute myocardial infarction	4.7 (1.3–11)	0.66 (0.25–1.0)
Systolic murmur increased with transient arterial occlusion	7.5 (2.5–23)	0.28 (0.13–0.60)
**Murmur intensity for detecting mitral regurgitation**		
**Murmur grade^α^**	**LR+ (95%CI) for severe regurgitation**	
4–6	14 (3.3–56)	
3	3.5 (2.1–5.7)	
0–2	0.19 (0.11–0.33)	
**Physical examination for detecting aortic regurgitation**		
	**LR+ (95%CI)**	**LR- (95%CI)**
Overall cardiac examination	5.1 (1.4–19)	0.82 (0.67–1.0)
S3	5.9 (1.4–25)	0.3 (0.73–0.95)
Popliteal-brachial gradient >20mmHg	8.2 (1.5–7.8)	0.2 (0.1–0.5)
Peripheral hemodynamic signs	2.1 (0.3–22)	0.8 (0.7–1.7)
Pulse pressure >50mmHg	.0 (0.7–2.2)	0.9 (0.2–5.5)
**Murmur intensity for detecting aortic regurgitation**		
**Murmur grade^α^**	**LR+ (95%CI) for severe regurgitation**	
4–6	4.5 (1.6–14)	
3	1.1 (0.5–2.4)	
0–2	0 (0–0.9)	

α Levine classification.

95%CI: 95% confidence interval; LR: likelihood ratio; S2: second split; S3: third split.

## ELECTROCARDIOGRAPHY AND CHEST RADIOGRAPHY

A complete electrocardiogram can now be performed rapidly at the bedside using increasingly portable equipment with reliable image transmission.^( 34 )^ Although chest radiography is not strictly a bedside test, it remains an easily accessible examination.^( 35 )^ These tests may reveal multiple changes associated with significant VHD. However, no comprehensive compilation of LR exists for each disease. In summary, left atrial overload combined with signs of pulmonary hypertension and right-sided chamber overload may suggest mitral stenosis. Left chamber overload is typically associated with MR. Left ventricular concentric hypertrophy may indicate AS, whereas left ventricular eccentric hypertrophy may suggest AR.

## ARTIFICIAL INTELLIGENCE

Artificial intelligence-driven medical technologies are developing rapidly into applicable solutions in clinical practice. Deep learning algorithms can process increasing amounts of data from various sources, yielding models that improve the diagnosis and management of different cardiac conditions.^( 36 )^ Regarding valvular disease, a recent study evaluated an electronic murmur detection algorithm in 603 outpatients, with a total of 3,180 heart sound recordings. The software identified pathologic cases with a sensitivity of 93% and a specificity of 81%, corresponding to a positive LR of 4.8 and a negative LR of 0.08.^( 37 )^ Likewise, an intelligent diagnostic system developed by Sun et al.^( 38 )^ discriminated AR, MR, and pulmonary stenosis with accuracies of 98.9%, 98.4%, and 98.7%, respectively. However, this emerging technology could not accurately recognize areas distant from the sternum.

In a retrospective study of more than 250,000 patients, Cohen-Shelly et al. identified moderate to severe AS in 3.7% of cases by echocardiography. Artificial intelligence analysis was applied to half of the patients using electrocardiograms, and further validation was conducted in 10% of the patients. The diagnostic performance showed a positive LR of 3 and a negative LR of 0.29 for AS.^( 39 )^ Another study using a similar methodology, but including external validation, reported a positive LR of 4.3 and a negative LR of 0.24 for artificial intelligence-based electrocardiogram analysis in the diagnosis of AS.^( 40 )^

Automatic detection of multiple diseases from chest imaging is a popular topic in radiology. However, only one study has examined the detection of VHD through analysis of the cardiovascular border on posteroanterior chest radiography. In this pilot study, the agreement between manual and software interpretation of border parameters was high, but current data are insufficient to support VHD diagnosis using this algorithm.^( 41 )^

Several studies have evaluated artificial intelligence in echocardiographic assessment, reporting high mean accuracy for automated software interpretation.^( 42 )^ Other studies have demonstrated that artificial intelligence algorithms can be applied across various modalities in POCUS.^( 43 )^

## POCUS

POCUS has been widely used, mainly over the last decade, as an extension of physical examinations. Its use is now well established in clinical practice for scenarios such as cardiac arrest, trauma, acute undifferentiated dyspnea, and shock.^( 44 , 45 )^ By assessing the lungs, left ventricular function, dynamic volume status parameters, and the abdominal cavity, POCUS provides physicians with a bedside tool that yields high positive and negative LR, consistently improving the diagnostic accuracy for the etiology of shock and hemodynamic disorders, while also saving time.^( 46 , 47 )^ However, in the outpatient setting of VHD, the usefulness and applicability of POCUS remain underexplored, and no specific algorithms have yet been proposed for clinical practice.

**Figure 3:**
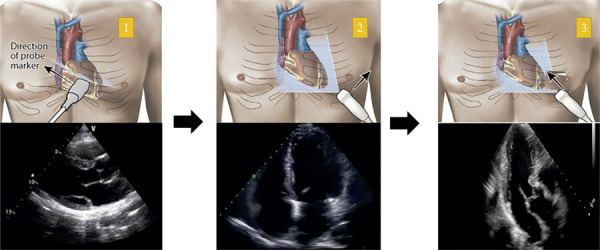
Echocardiographic views and probe positioning for the ValvUS protocol

Source: Aly I, Rizvi A, Roberts W, Khalid S, Kassem MW, Salandy S, et al. Cardiac ultrasound: an anatomical and clinical review. Transl Res Anat. 2021;22:100083.^(^ ^52^ ^)^

**Figure 4:**
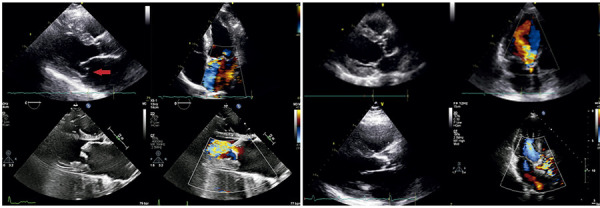
POCUS assessment of mitral and aortic valve disease

Standard echocardiographic evaluation of patients with VHD is complex. It includes several qualitative and quantitative criteria, making bedside assessment challenging and requiring simplification into a few objective points to be feasible.^( 3 )^ The main criteria used to assess VHD severity include: i) valvular morphology, valve area, and transvalvular pressure gradient, which determine flow turbulence visualized as color Doppler aliasing; ii) left atrial and ventricular dimensions; iii) left ventricular function; and iv) assessment of the regurgitant jet, mainly its width, in cases of valve regurgitation.^( 1 , 47 - 51 )^ A POCUS approach should first assess these anatomic criteria of valvular severity, followed by evaluation of the hemodynamic repercussions.^( 48 )^ Adapting standard echocardiographic recommendations to bedside feasibility, we propose a focused valvular ultrasound approach (ValvUS protocol) comprising five simple steps: evaluation of valve leaflet motion, thickness, regurgitant flow analysis, detection of aliasing, and visual estimation of chamber size and function. All criteria can be evaluated using only three echocardiographic views: the parasternal long-axis view and the apical four- and three-chamber views (Figures 3 and 4 ). It is important to emphasize that the ValvUS approach should be integrated with the traditional clinical pre-test probability to achieve the most accurate bedside diagnosis. The greater the number of consistent clinical and ultrasound findings, the higher the positive LR for severe VHD – and conversely, fewer findings reduce diagnostic certainty. It should be noted that the ValvUS approach has not yet been extensively validated. Future studies across various VHD scenarios are needed to further evaluate the role of POCUS and its associated LR in each valvular condition. As illustrated in the central figure, we believe that, when combined with clinical suspicion, physical examinations, and emerging artificial intelligence technologies, POCUS may become an important bedside tool for identifying severe VHD in the near future.

Mitral Valve: Mitral regurgitation: A) parasternal long-axis view - systolic displacement of the mitral leaflets into the left atrium (red narrow) with associated left atrial enlargement; B) parasternal long-axis view - regurgitant flow from the left ventricle to the left atrium during systole. Mitral stenosis: C) parasternal long-axis view - thickening and calcification of the mitral valve with left atrial enlargement; D) parasternal long-axis view - flow aliasing observed distal to the mitral valve.

Aortic Valve: Aortic regurgitation: A) parasternal long-axis view - thickened aortic valve with marked eccentric left ventricular hypertrophy; B) apical three-chamber view - color Doppler demonstrating aortic regurgitation from the aorta to the left ventricular apex during diastole (red narrow). Aortic stenosis: C) parasternal long-axis view - thickening and calcification of the aortic valve with concentric or eccentric left ventricular hypertrophy; D) apical three-chamber view - flow aliasing distal to the aortic valve. Laminar flow appears as a uniform blue color before the aortic valve, whereas after the stenotic region, an acceleration pattern is observed on color Doppler, with a mosaic of blue, red, and yellow signals.

## COMMENTS

The physical examination skills of clinicians are insufficient for an accurate diagnosis of severe VHD; however, they usually initiate the diagnostic flow. Electrocardiography and chest radiography, which are easily obtained, may contribute to the diagnosis of severe VHD by demonstrating left-sided chamber remodeling. This process may soon be enhanced by artificial intelligence-based analyses in clinical practice. Importantly, extending the physical examination with POCUS is fundamental in many aspects of patient assessment. The proposed ValvUS approach, which evaluates five components (valve leaflet motion, thickness, regurgitant flow, aliasing, and chamber dimensions), may also assist in identifying severe VHD at the bedside. This information needs to be integrated with other clinical features, as traditional ultrasonographic findings can increase the pre-test probability and thereby prompt an urgent comprehensive echocardiographic assessment. Future studies with large patient cohorts are required to confirm the overall role of POCUS in clinical practice and to establish its diagnostic accuracy across various VHD pathologies.

DATA AVAILABILITY:

The underlying content is contained within the manuscript.
